# Evolution of the nutritional quality of packaged food supply in low- and middle-income countries following the implementation of a front-of-pack labeling scheme

**DOI:** 10.3389/fnut.2025.1636713

**Published:** 2025-11-13

**Authors:** Marie Tassy, Andreas Rytz, Alison L. Eldridge, Tsz Ning Mak, Frank Ekow Atta Hayford, Edith J. M. Feskens

**Affiliations:** 1 Division of Human Nutrition and Health, Wageningen University & Research, Wageningen, Netherlands; 2 Nestlé Institute of Health Sciences, Nestlé Research, Société des Produits Nestlé, Lausanne, Switzerland; 3 Nestlé Institute of Agricultural Sciences, Nestlé Research, Société des Produits Nestlé, Lausanne, Switzerland; 4 Department of Dietetics, School of Biomedical and Allied Health Sciences, College of Health Sciences, University of Ghana, Accra, Ghana

**Keywords:** food supply, nutrient content, low- and middle-income countries, front-of-pack labeling, nutrient profiling

## Abstract

**Introduction:**

As packaged foods consumption increases in low- and middle-income countries (LMICs), certain governments have introduced front-of-pack labeling (FOPL) schemes to promote healthier food choices. This study assesses the evolution and nutritional quality of packaged foods in LMICs from 2015 to 2023 and examines trends in countries where FOPLs have been implemented.

**Methods:**

On-pack information from products in the top 20 packaged food categories was retrieved from the Mintel Global New Product Database (2015–2023) in 19 LMICs. The number of new products introduced and median content of energy, sugars, sodium, saturated fatty acids (SFA), protein, and fiber were analyzed by product category, country, region and type of FOPL implemented. Evolution of the percentage of products with an improved nutritional content was compared from 2015–2017 to 2021–2023.

**Results:**

Our findings indicate that from 2015 to 2023, the percentage of packaged meat and coffee products increased in LMICs, while more indulgent products such as cookies declined. The nutritional quality of products improved, particularly toward a reduction in total sugars and an increase in protein content. The implementation of FOPL was associated with further reductions in total sugars, and, depending on the type of scheme implemented, with reduction in sodium.

**Discussion:**

These findings offer insights on the food environment in LMICs undergoing a nutrition transition, and on how certain food policies can be associated with reformulation of packaged foods in those countries.

## Introduction

Over the last decades, low- and middle-income countries (LMICs) have faced a double burden of malnutrition, characterized by the coexistence of undernutrition, micronutrient deficiencies and overnutrition (1). The rise in overweight and obesity prevalence is, at least in part, due to a change in the food system. Packaged foods and beverages, that are industrially pre-prepared and sealed to be suitable for retail sale, are becoming more available and affordable (2, 3) leading to a transition from traditional to more westernized diets (4). As this phenomenon also known as the nutrition transition occurs, high fiber, low fat products are being replaced with animal-based and packaged foods, that can be higher in sodium, fats and sugars (5).

To encourage the consumption of healthier foods, 16 LMICs had implemented food policies such as front-of-pack labeling (FOPL) schemes, food taxes, and claims regulations (6). Those policies are supported by nutrient profiling models (NPMs) which enable the “classification or ranking foods according to their nutritional composition for reasons related to preventing disease and promoting health” (7). Most NPMs rank foods according to their content in fats, sugars and salt. Additionally, some NPMs include micronutrients to address the issue of malnutrition such as the Healthier Choice logo in Malaysia and Thailand. Other models include whole grains and fiber in countries where overnutrition is highly prevalent (6).

Most NPMs in LMICs support FOPL schemes to improve consumer awareness in making healthier food choices (8). FOPL provides clear and concise nutritional information on-pack and therefore mitigates information asymmetry between consumers and suppliers, addressing the challenge of quality uncertainty that consumers face when making purchasing decisions (9). Additionally, FOPL encourages reformulation of packaged foods toward levels of nutrients aligned with dietary guidelines (8). FOPL represents a cost–benefit response to labeling regulations for the food industry as it can enhance market positioning and build consumer trust. Ultimately, FOPL not only empowers consumers with better information but also encourages firms to maintain higher quality standards. In LMICs, implementation of FOPL schemes has been efficient and has positively impacted consumer awareness in making healthier food choices. Brazilian High In labels were widespread on packaged foods in top consumed categories 1 year after their adoption (10). Peruvian High In labels were efficient in informing consumers from different settings and socio-economic status on the healthfulness of packaged products (11). Ecuadorian Traffic Lights were understood by most participants in a recent study; however, this did not always lead to a change in attitude and practices (12). Except in Peru and Mexico where High In labels have encouraged reformulation of critical nutrients, particularly energy and saturated fats (13–15), the impact of FOPL on reformulation of packaged foods in LMICs remains unclear.

In fact, new product introductions and nutritional quality of the packaged food supply in LMICs have rarely been evaluated. Some analyzes were performed cross-sectionally and at a country level. Ndanuko et al. studied the sodium content of more than 6,000 packaged products in Kenya in 2019 and compared it with the South-African food supply finding wide variabilities within categories (16). Similarly, Pongutta et al. observed that only 9% of ready-to-eat packaged foods in 2015 in Thailand were classified as healthier according to the Thai nutrient profiling system (17). On the global scale, newly launched packaged foods were significantly lower in total sugars and sodium in 2020 than in 2016 (18), but this study did not evaluate changes in the packaged food supply by country’s level of income. Therefore, it is unclear if the same trends are observed across LMICs.

The aim of this study was to assess the evolution of the nutritional quality of the packaged food supply in LMICs and to evaluate if trends have been any different after the implementation of a FOPL in each country. This study focused on packaged foods and food policies targeting the general population, therefore products specific to infants and children are not considered.

## Methods

### Food supply database

The number of new products introduced, and the nutritional quality of packaged foods was characterized by performing a secondary analysis of the Mintel Global New Product Database (GNPD) (19). Mintel GNPD is a commercial repository of new packaged food products launched globally. It reports all information available on-pack in 141 categories and across 81 countries and is used to monitor claims and nutrient content of packaged products (20–22).

A subset of Mintel GNPD featuring packaged food products launched from 2015 to 2023 in 19 LMICs was extracted. Countries were classified using the World Bank 2023 classification by region and income (23). A LMIC was selected if information was available for more than 1,000 products every year in Mintel GNPD (Table 1). The presence or absence of a FOPL scheme was determined based on a recent systematic review of nutrient profiling models supporting food policies in LMICs (6). Three FOPL have been implemented in the countries considered: High In labels, Traffic Light schemes and Choices schemes. High In labels are a type of mandatory FOPL implemented across most Latin American LMICs and more recently in South Africa. They convey negative messaging by framing in black nutrients present in excess (12). Traffic Lights schemes are implemented in Ecuador and Sri Lanka and deliver a mixed message by coloring nutrient in red if their content is high, yellow if moderate and green if low (6). The Choices scheme, implemented mainly in South Asia, highlights the healthiest options per food category with a logo (6). Since High In labels were implemented in South Africa in 2023 (24), they could not have had an impact on nutritional quality from 2015 to 2022 and therefore South Africa was not considered in this analysis.

**Table 1:** List of LMICs considered with their region, front-of-pack labeling scheme and number of products.

Region	Market	ISO	Front-of-pack labeling scheme	Year of implementation	*N* products
Latin America	Peru	PER	High in labels	2017	7,146
Latin America	Brazil	BRA	High in labels	2020	39,103
Latin America	Mexico	MEX	High in labels	2020	23,392
Latin America	Colombia	COL	High in labels	2022	14,750
Latin America	Argentina	ARG	High in labels	2022	13,976
Latin America	Ecuador	ECU	Traffic lights	2014	6,080
South Asia	Sri Lanka	LKA	Traffic lights	2019	8,053
East Asia	Thailand	THA	Choices	2016	15,928
East Asia	Malaysia	MYS	Choices	2017	7,193
East Asia	Indonesia	IDN	Choices	2019	22,582
East Asia	Vietnam	VNM	No FOPL		15,948
East Asia	Philippines	PHL	No FOPL		8,483
East Asia	China	CHN	No FOPL		44,435
Europe and Central Asia	Turkey	TUR	No FOPL		7,406
Middle East and North Africa	Egypt	EGY	No FOPL		6,708
Middle East and North Africa	Morocco	MAR	No FOPL		5,371
South Asia	India	IND	No FOPL		50,332
Sub-Saharan Africa	South Africa	ZAF	No FOPL		15,639
Sub-Saharan Africa	Nigeria	NGA	No FOPL		14,669

Food categories were selected based on the percentage of products per category for each LMIC and averaged across countries. Only the top 20 categories were selected for further analysis (Table 2). These foods represent each more than 1.5% of the food supply summing to nearly half of newly launched packaged foods in LMICs. A total of 327′194 packaged food products were included in the final dataset.

**Table 2:** List of packaged food product categories and respective number of products.

SubCategory	East Asia	Latin America	South Asia	Sub-Saharan Africa	Middle East and North Africa	Europe and Central Asia	All regions
Baking ingredients	2,897	7,483	5,650	2,202	680	455	19,367
Bread	3,622	7,456	2,619	878	316	250	15,141
Cakes	12,175	6,510	1,486	1,396	508	240	22,315
Carbonated Drinks	3,053	3,035	1,194	1,363	460	233	9,338
Cereals	2,734	4,537	1,312	1,175	788	205	10,751
Coffee	5,673	4,933	1736	917	974	311	14,544
Cookies	15,052	12,652	4,826	4,956	1,582	601	39,669
Fish	6,795	4,367	898	1,319	759	277	14,415
Fruit Snacks	7,986	2,884	2,729	1,088	324	221	15,232
Meat	8,410	7,282	273	1,441	404	827	18,637
Nuts	7,447	3,584	5,079	1,124	254	450	17,938
Oils	3,529	3,581	3,328	1,160	706	570	12,874
Pasta	961	5,651	915	812	708	302	9,349
Poultry	3,686	3,273	1,111	1,033	571	339	10,013
Rice	4,415	2,259	2,802	534	499	285	10,794
Seasonings	6,256	7,235	10,798	3,571	539	428	28,827
Still Drinks	4,546	2,619	1,649	841	338	168	10,161
Table Sauces	5,096	4,659	1,163	1,003	335	260	12,516
Tea	6,904	4,271	4,253	1767	755	383	18,333
Vegetables	3,332	6,176	4,564	1728	579	601	16,980
All categories	114,569	104,447	58,385	30,308	12,079	7,406	327,194

**Figure 1:**
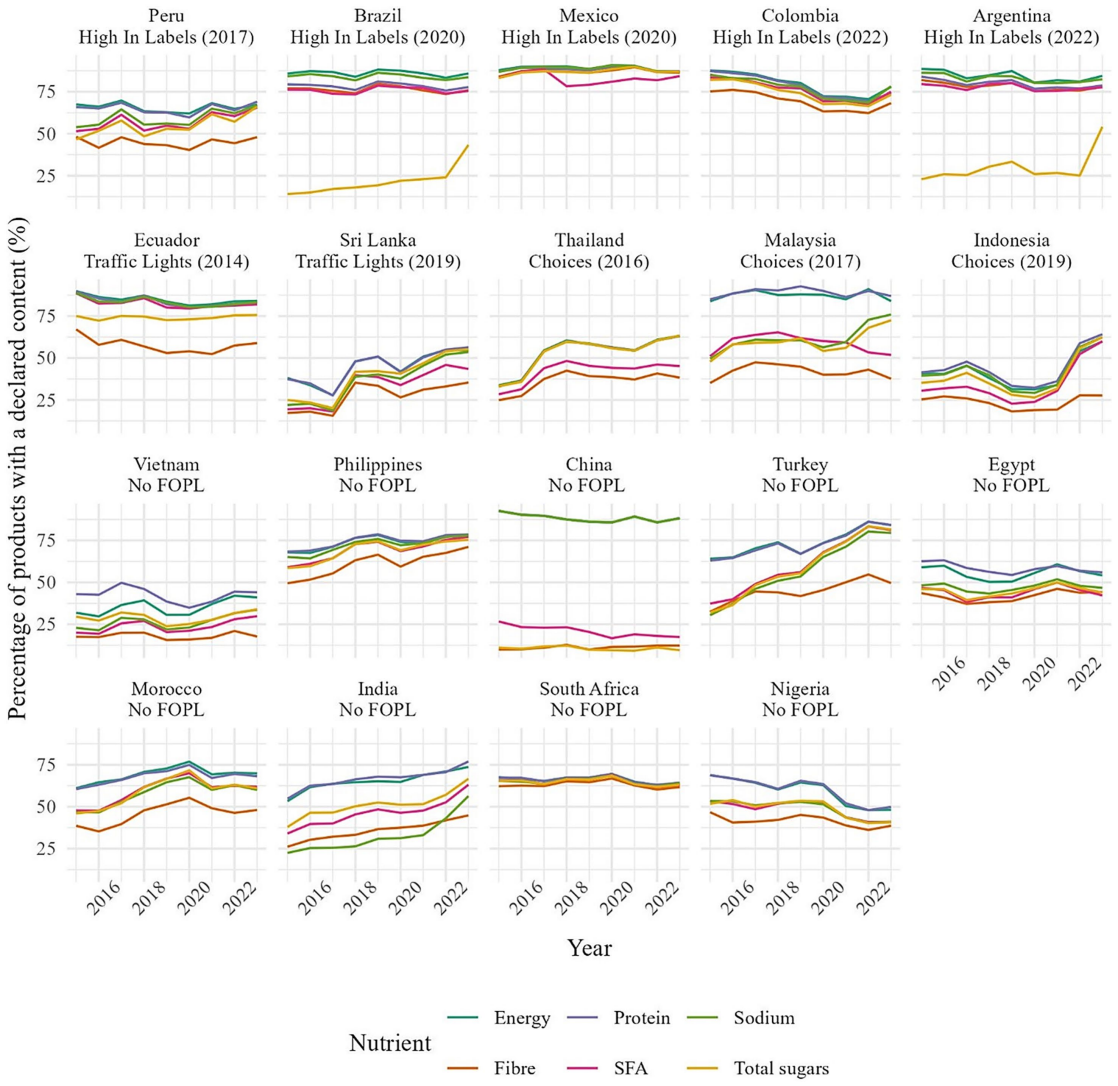
Percentage of products with a declared content in energy, SFA, sugars, sodium, protein and fiber by country.

For each product considered, information was extracted on declared content of energy, total sugars, sodium, saturated fatty acids (SFA), protein and fiber. Those nutrients were selected because they are relevant to public health, included in most nutrient profiling models (25, 26) and mandatory to declare in a majority of countries (27). However, the rate of declaration was variable per country and per nutrient (Figure 1). In Argentina and Brazil, less than 30% of packaged foods declare content of total sugars because declaration was not mandatory in those two countries before 2021/2022 (28–30). In China, only sodium is declared in more than 80% of products despite existing regulations (31). Variation in the number of products with a declared nutrient content by country has been taken into account in the statistical analyses.

Data extracted from the Mintel GNPD was split into three periods of 3 years: from 2015 to 2017, from 2018 to 2020 and from 2021 to 2023. Each period featured more than 100,000 products, enough products to analyze the evolution of food supply at a country, region and category level.

### Statistical analysis

Change of the number of products in each food category and the nutritional quality of the packaged food supply was evaluated by comparing the number and median nutrient content of the five nutrients of interest by country and category, in 2015–2017 and 2021–2023. Median nutrient content was selected over mean to give more importance to central values than to extreme ones in each category. Percentage of products and median nutrient content by category were averaged across countries to identify category-specific changes. A t-test was used for comparison between the two time periods, with Benjamini-Hochberg (BH) correction to account for multiple testing (*α* = 5%).

Country-specific changes in number of products and nutritional quality were mapped using Principal Components Analysis (PCA) in 2015–2017 and in 2021–2023. Countries were colored by regions to identify regional patterns.

In addition, change in nutrient content was assessed by difference-in-difference analysis in countries where a FOPL has been implemented. The percentage of products with an “improved” nutrient content in 2018–2020 and 2021–2023 was compared to the 2015–2017 median value for each category within each country to identify improvements in nutrient content independently of the baseline value and by type of FOPL. Percentages were then aggregated at a country level with a 95% confidence interval. “Improved” was defined as lower content than the 2015–2017 median for energy, total sugars, SFA and sodium and higher content than the 2015–2017 median for protein and fiber. Chinese products as well as total sugars in Brazilian and Argentinian products were excluded from the analysis due to inconsistent declaration rates over the years (Figure 1). Only categories with a non-null median were considered.

Changes in nutrient content were aggregated by type of FOPL to assess the impact of this food policy across countries. Percentages of products with an “improved” nutrient content as well as 95% confidence interval were aggregated by type of FOPL implemented in the country, i.e., High in labels, Traffic Lights or Choices schemes. Nutrient content in 2018–2020 and 2021–2023 was compared to country-specific and category specific medians in 2015–2017 to assess nutritional improvement irrespective of baseline values which may be affected by local regulatory and socio-economic factors.

## Results

### Evolution of the composition of the food supply

From 2015–2017 to 2021–2023, the composition of the food supply has evolved while the total number of products launched in the two periods of time remained steady. The percentage of new launches in four product categories decreased by more than 1 percentage point: Cakes (−1.0), Cookies (−2.6), Still Drinks (−1.2) and Vegetables (−1.5) while it increased by more than 1 percentage point in Coffee (+2.2), Meat (+2.0) and Poultry (+1.0) (Table 3).

**Table 3:** Average median content of energy, saturated fatty acids, sodium, total sugars, fiber and proteins in top 20 categories across countries in 2015–2017 and average absolute differences with 2021–2023.

Product category	Composition of the food supply					Energy					
	N products 2015–2017	% products 2015–2017	N products 2021–2023	% products 2021–2023	Difference in percentage	N products 2015–2017	N products 2021–2023	Average median content 2015–2017 (kcal/100 g)	Average absolute difference in 2021–2023 (kcal/100 g)	Average percentage difference in 2021–2023 (kcal/100 g)	adjusted p-value
**All**	**110,052**	/	**109,296**	/		**76,500**	**78,324**	**353**	**−10**	**−3%**	**0.07**
Baking Ingredients	6,599	6%	6,433	6%	−0.1%	4,447	4,871	361	−3	−1%	0.17
Bread	5,312	5%	4,963	5%	−0.3%	4,108	4,314	304	−3	0%	0.67
Cakes	8,213	7%	7,116	7%	−1.0%	5,716	5,774	400	−8	−2%	0.06
Carbonated Drinks	3,249	3%	3,148	3%	−0.1%	2,877	2,924	39	−12	−31%	0.00
Cereals	3,329	3%	3,545	3%	0.2%	3,143	3,300	386	8	2%	0.02
Coffee	3,738	3%	6,121	6%	2.2%	1,671	2,701	394	−19	−4%	0.36
Cookies	14,785	13%	11,849	11%	−2.6%	12,536	10,521	483	2	0%	0.40
Fish	4,719	4%	5,146	5%	0.4%	3,260	3,582	152	1	2%	0.86
Fruit Snacks	5,288	5%	4,467	4%	−0.7%	4,010	3,396	337	−9	−1%	0.32
Meat	4,961	5%	7,153	7%	2.0%	2,981	4,462	230	8	4%	0.39
Nuts	5,742	5%	6,222	6%	0.5%	4,350	4,786	589	0	0%	0.89
Oils	3,842	3%	4,850	4%	0.9%	3,412	4,249	862	−3	0%	0.46
Pasta	3,549	3%	2,753	3%	−0.7%	3,386	2,624	354	−8	−2%	0.38
Poultry	2,786	3%	3,913	4%	1.0%	1936	2,496	188	−5	−2%	0.19
Rice	3,465	3%	3,508	3%	0.1%	2,341	2,379	349	−11	−3%	0.34
Seasonings	9,951	9%	9,700	9%	−0.2%	3,668	3,980	220	22	11%	0.38
Still Drinks	3,976	4%	2,606	2%	−1.2%	3,464	2,349	42	−8	−21%	0.00
Table Sauces	3,890	4%	4,684	4%	0.8%	3,020	3,622	114	3	5%	0.56
Tea	5,983	5%	6,105	6%	0.1%	2,360	2,597	92	31	17%	0.09
Vegetables	6,675	6%	5,014	5%	−1.5%	3,814	3,397	102	28	33%	0.07

Product category	Saturated fatty acids						Sodium					
	*N* products 2015–2017	*N* products 2021–2023	Average median content 2015–2017 (g/100 g)	Average absolute difference in 2021–2023 (g/100 g)	Average percentage difference in 2021–2023 (g/100 g)	adjusted *p*-value	*N* products 2015–2017	*N* products 2021–2023	Average median content 2015–2017 (kcal/100 g)	Average absolute difference in 2021–2023	Average percentage difference in 2021–2023 (mg/100 g)	adjusted *p*-value
All	54,706	59,862	1.6	0.3	32%	0.23	65,485	71,673	183	−3	−4%	0.80
Baking Ingredients	3,279	3,987	0.9	−0.1	−28%	0.62	3,522	4,171	180	−12	21%	0.69
Bread	3,249	3,269	1.7	0.4	27%	0.04	3,474	4,060	435	−27	−5%	0.07
Cakes	3,489	3,057	7.2	−0.4	−5%	0.42	5,030	5,545	206	6	7%	0.57
Carbonated Drinks	2,383	2,556	0.0	0.0	NaN	NaN	2,497	2,716	7	1	13%	0.19
Cereals	2,862	2,887	1.0	0.3	33%	0.03	2,928	3,175	252	−75	−26%	0.00
Coffee	1,097	1839	5.5	−0.1	−3%	0.90	1,348	2,305	141	−1	5%	0.97
Cookies	8,905	8,110	10.4	0.3	4%	0.33	10,756	9,967	220	1	2%	0.90
Fish	2,254	2,896	1.4	−0.1	19%	0.71	2,990	3,545	516	−103	−8%	0.24
Fruit Snacks	2,691	2,422	0.0	0.1	−73%	0.26	3,670	3,152	27	−2	50%	0.79
Meat	1903	3,414	5.5	0.5	2%	0.51	2,787	4,398	892	−143	−9%	0.06
Nuts	2,113	3,014	6.7	−0.3	−4%	0.46	3,855	4,365	320	−49	−13%	0.09
Oils	2,802	3,777	14.4	1.1	7%	0.26	2,517	3,218	0	0	NaN	NaN
Pasta	2,865	2,431	0.3	0.0	−12%	0.59	2,924	2,430	6	21	733%	0.32
Poultry	1,342	1812	3.1	−0.2	−2%	0.39	1,513	2,372	574	−27	20%	0.53
Rice	1,520	1739	0.1	0.0	8%	0.88	1985	2004	7	−4	378%	0.27
Seasonings	2,398	3,008	0.3	0.3	32%	0.22	3,024	3,498	5,094	−240	−5%	0.83
Still Drinks	2,872	1967	0.0	0.0	NaN	0.33	2,879	2052	11	0	−5%	0.71
Table Sauces	2,219	2,930	0.1	0.0	7%	0.69	2,681	3,588	1,662	−93	−4%	0.52
Tea	1,601	1947	0.0	0.5	NaN	0.33	1,615	1993	11	6	0%	0.26
Vegetables	2,862	2,800	0.0	0.0	101%	0.05	3,490	3,119	136	−10	25%	0.59

Product category	Total sugars						Fiber					
	*N* products 2015–2017	*N* products 2021–2023	Average median content 2015–2017 (kcal/100 g)	Average absolute difference in 2021–2023	Average percentage difference in 2021–2023 (g/100 g)	Adjusted *p*-value	*N* products 2015–2017	*N* products 2021–2023	Average median content 2015–2017 (kcal/100 g)	Average absolute difference in 2021–2023	Average percentage difference in 2021–2023 (g/100 g)	Adjusted *p*-value
All	43,871	53,279	7.8	−2.0	−19%	0.05	48,009	51,550	2.0	0.0	0%	0.71
Baking Ingredients	2062	3,156	15.8	−9.5	−33%	0.00	3,361	3,825	2.9	0.6	20%	0.17
Bread	2,187	2,508	4.4	0.4	50%	0.35	3,163	3,153	3.3	0.0	0%	0.97
Cakes	2,637	2,779	26.2	−0.6	14%	0.71	3,177	2,694	1.6	0.6	39%	0.01
Carbonated drinks	2,260	2,611	9.2	−3.0	−32%	0.00	977	1,248	0.0	0.0	NaN	NaN
Cereals	2,670	2,704	25.0	−3.6	−14%	0.00	3,028	3,144	5.7	0.7	19%	0.02
Coffee	1,020	1775	26.5	−5.0	36%	0.19	836	1,230	3.9	0.8	328%	0.63
Cookies	7,897	7,453	28.7	−1.4	−5%	0.19	7,638	6,724	2.6	0.4	12%	0.03
Fish	2,185	2,802	0.3	0.0	0%	0.88	2,162	2,609	0.1	0.0	−56%	0.82
Fruit Snacks	1,478	1851	40.5	3.4	89%	0.44	1817	2017	5.3	0.3	8%	0.15
Meat	1,633	3,048	1.2	−0.6	62%	0.37	1845	2,913	0.4	−0.1	−48%	0.37
Nuts	1713	2,851	5.1	0.6	32%	0.55	2,184	3,043	7.2	0.4	7%	0.24
Oils	3,114	3,820	0.0	0.0	NaN	NaN	3,100	3,767	0.0	0.0	NaN	NaN
Pasta	1700	1725	2.5	−0.1	12%	0.76	2,858	2,346	3.0	0.2	9%	0.10
Poultry	1,192	1779	0.6	−0.2	−37%	0.08	1,212	1,479	1.1	−0.1	−31%	0.42
Rice	945	1,339	0.3	−0.1	−9%	0.51	1,557	1741	1.5	0.4	31%	0.22
Seasonings	2,203	2,934	3.6	−0.5	−4%	0.42	2,236	2,651	1.9	1.6	251%	0.07
Still drinks	2,230	1,668	9.0	−2.3	−27%	0.00	1,319	866	0.1	0.0	−56%	0.73
Table Sauces	1731	2,628	10.3	0.8	20%	0.42	1,443	1904	0.3	0.1	6%	0.26
Tea	1,294	1768	1.7	3.7	345%	0.14	1,090	1,321	0.0	0.4	208%	0.19
Vegetables	1720	2080	1.8	0.2	29%	0.48	3,006	2,875	3.4	2.4	74%	0.01

Product category	Protein					
	*N* products 2015–2017	*N* products 2021–2023	Average median content 2015–2017 (kcal/100 g)	Average absolute difference in 2021–2023	Average percentage difference in 2021–2023 (g/100 g)	adjusted p-value
All	76,155	77,610	6.5	0.4	7%	0.01
Baking Ingredients	4,455	4,870	7.2	0.6	11%	0.17
Bread	4,154	4,338	9.2	−0.2	−1%	0.49
Cakes	5,815	5,845	5.7	0.2	3%	0.07
Carbonated Drinks	2,406	2,584	0.0	0.0	NaN	0.33
Cereals	3,166	3,337	7.8	0.4	6%	0.01
Coffee	1,612	2,676	7.3	0.9	19%	0.35
Cookies	12,612	10,701	6.4	0.0	0%	0.81
Fish	3,441	3,642	17.1	0.7	−1%	0.37
Fruit Snacks	4,025	3,403	2.2	0.2	13%	0.20
Meat	3,225	4,530	15.1	0.3	0%	0.68
Nuts	4,425	4,856	19.4	−0.1	0%	0.84
Oils	3,117	3,842	0.0	0.0	NaN	NaN
Pasta	3,395	2,653	11.7	0.0	1%	0.94
Poultry	1991	2,530	13.7	1.1	2%	0.23
Rice	2,413	2,424	7.1	−0.2	−3%	0.33
Seasonings	3,655	3,952	5.3	0.8	−2%	0.39
Still Drinks	3,205	2,116	0.0	0.0	185%	0.76
Table Sauces	2,779	3,344	1.3	0.0	2%	0.89
Tea	2,380	2,553	1.4	−0.1	−19%	0.77
Vegetables	3,884	3,414	4.8	1.7	24%	0.14

Average median content is the average of median content in energy or nutrient in each country. The average absolute difference is the average difference between the median content in energy or nutrient in 2015–2017 and 2021–2023. *p*-value of a *t*-test adjusted with Benjamini-Hochberg (BH) method. Adjusted *p*-values < 0.05 are highlighted in green. Bold values indicates results for all categories together, in opposite to other rows that are category-specific.

**Figure 2:**
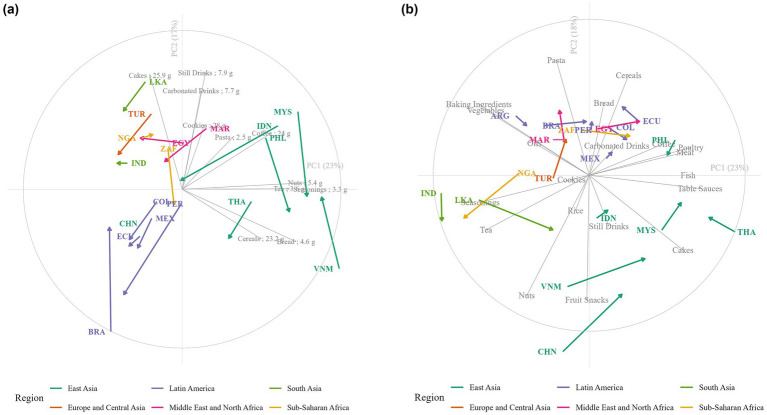
Principal component analysis of **(a)** the food supply and **(b)** the median content in sugars of packaged foods in top 20 categories in LMICs. Country-specific change from period 2015–2017 to 2021–2023 is indicated with an arrow. Countries are labeled with their ISO code. In **(b)**, product categories’ names are followed by the average median content in total sugars in grams per 100 grams in the product category.

LMICs were mapped according to the top 20 product categories in their food supply using PCA (Figure 2a). Changes in the newly launched food supply from the period 2015–2017 to 2021–2023 are represented with arrows. Food supply from countries in the same region are grouped together and most arrows are short except for Sri Lanka, Nigeria Vietnam and China suggesting that the composition of the food supply remains regionalized over time. Additionally, East Asian newly launched packaged foods belonged mostly to animal-based categories (Poultry, Meat, Fish), as well as Table Sauces, and Snacks. In India, Sri Lanka, and in Nigeria, most launches happened in staples and ingredients (Seasonings, Tea, Baking Ingredients, Vegetables). Latin American and Mediterranean countries relied mostly on wheat-based products such as Pasta, Bread, and Cereals.

### Evolution of the nutritional quality of the food supply

Newly launched packaged foods had significantly lower content in total sugars in 2021–2023 than in 2015–2017. Median content in total sugars decreased by 2.0 g/100 g (−19%) on average over the past 9 years across categories, in particular Baking Ingredients (−9.5 g/100 g; −33%), Carbonated Drinks (−3 g/100 g; −32%), Breakfast Cereals (−3.6 g/100 g; −14%) and Still Drinks (−2.3 g/100 g; −27%) (Table 3). Country-specific evolution in total sugars content is represented with an arrow in Figure 2b. Most arrows except for Brazil, Egypt, Nigeria and Vietnam are directed toward the bottom or the left corner which demonstrates that the reduction in total sugars is observed across most countries. Longer arrows such as for Malaysia, Philippines, Indonesia and Peru suggest larger reductions in those countries. Additionally, median total sugars content varied by region. New launches were lower in total sugars content in sweet product categories such as Carbonated and Still Drinks or Cookies in Latin America than in other regions (Figure 2b). The East Asian food supply was higher in total sugars in savory categories such as Seasonings, Bread and Nuts than any other region.

Packaged food launches were significantly higher in protein content in 2021–2023 than in 2015–2017. Median protein content increased by 7% between the two periods, particularly in breakfast Cereals (+0.4 g/100 g) (Table 3). No pattern could be observed across regions using PCA.

No significant change nor pattern could be observed in energy, SFA, sodium or fiber across categories (Table 3). However, there were changes at the category level. Energy content dropped by −31% and −21% in Carbonated and Still Drinks, respectively, in 2021–2023. Breakfast Cereals had a lower content of sodium (−26%) and total sugars (−14%) but a higher content of energy (+2%), SFA (+33%), protein (+6%) and fiber (+19%). Fiber content also increased in Cakes (+0.6 g/100 g), Cookies (+0.4 g/100 g) and in packaged Vegetables (+2.4 g/100 g) (Table 3).

### Association with front-of-pack labeling: difference-in-difference analysis

**Figure 3:**
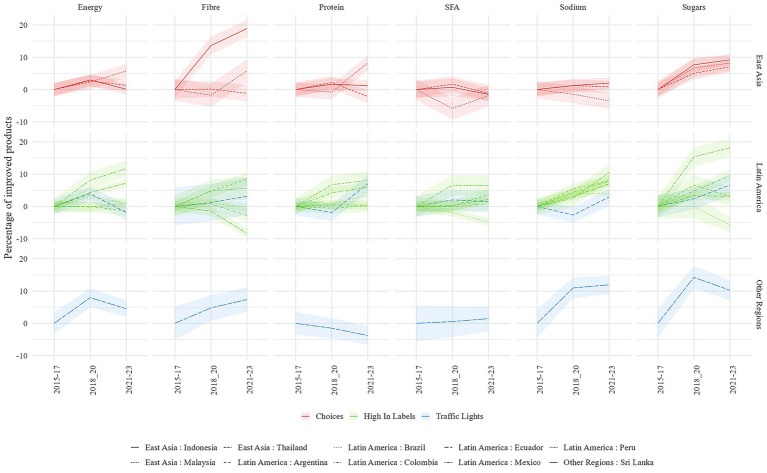
Difference in the percentage of products having an improved median content in energy, fiber, saturated fatty acids, protein, sodium and total sugars compared with 2015–2016-2017 by country and region. Only countries with a FOPL implemented are presented. Ribbon represents confidence interval at 95%. Only categories with a non-null median are considered in this analysis.

The type of FOPL is region-specific, with Choices in South East Asia, High In Labels for most Latin American countries except Ecuador and one LMIC, Sri Lanka, in other regions. The percentage of products with a lower total sugars content than the 2015–2017 median increased in all LMICs with a FOPL, except for Brazil (Figure 3). Regional trends could also be observed. In East Asian countries, the percentage of products with a sugar content higher than the 2015–2017 median increased by more than 5% in 2021–2023. In Latin America, the percentage of products with a lower sodium content increased by 1–9% in 2021–2023. Some countries have also performed better than others in the same region. In Latin America, the biggest improvement for energy, protein, SFA and total sugars happened in Peru, the first LMIC in which High In labels were implemented (Figure 3).

**Figure 4:**
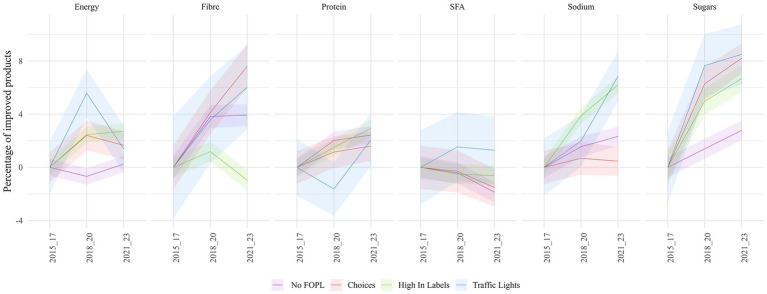
Difference in the percentage of products having an improved median content in energy, fiber, saturated fatty acids, protein, sodium and total sugars compared with 2015-2016-2017 by front-of-pack labeling. Ribbon represents confidence interval at 95%. Only categories with a non-null median are considered in this analysis.

The number of products having an improved median content of energy and nutrients compared to the 2015–2017 median varied depending on the type of FOPL implemented. In 2021–2023, more than 55% of products had a total sugars content lower than 2015–2017 median in countries with a FOPL. This is significantly higher than in countries without FOPL (52%) and was true regardless of the type of FOPL implemented (Figure 4). The percentage of products with a sodium content lower than the 2015–2017 median increased from 49 to 55% in countries with High In labels and from 48 to 55% in countries with Traffic Lights, while it did not increase to more than 51% in other countries. The percentage of products with an improved content in fiber increased significantly from 46 to 54% in countries with the Choices label, from 46 to 53% in countries with Traffic Lights, from 48 to 52% in countries with no FOPL but did not change significantly in countries with High In labels. No patterns could be observed for energy and SFA (Figure 4).

## Discussion

This study is the first to assess the changes in product offerings and nutritional quality of the packaged food supply in 19 LMICs across continents over 9 years, and to describe how trends have changed after the implementation of a FOPL. From 2015 to 2023, the number of animal-based and coffee products launched has increased, while fewer sweet products such as cookies, cakes, and soft drinks have been introduced. This shift in product offerings, together with category-specific improvements in the nutritional quality of packaged foods, are correlated with a reduction in total sugars content and an increase in protein content of newly launched foods in LMICs. Additionally, in countries where a FOPL has been implemented, there was a further reduction in total sugars content and, where High In labels or Traffic Lights were used, a reduction in sodium content.

Over the 9 years studied, the proportion of Poultry and Meat increased, respectively, by 1 and 2 percentage points, but the protein content did not change significantly in most product categories except for breakfast cereals. This suggests that the overall increase in protein content in LMICs newly launched packaged food supply could be due to a change in the increased number of products on the market rather than to reformulation. There are some exceptions such as Peru where protein content has increased in newly-launched products. This global rise in animal-based products consumption in LMICs has previously been described in the scientific literature as the protein transition. As LMICs consumers transition from traditional to westernized diets, the main sources of protein in their diet are not staple grains, pulses, and root crops anymore but meat, eggs and dairy (32, 33). This shift in protein consumption primarily results from economic growth and increased income levels and leads to higher demand for livestock in LMICs (34).

The proportion of newly launched Cookies, Cakes and Still Drinks has reduced by at least 1 percentage point from 2015 to 2023. In addition, median content in total sugars decreased by 2.0 g/100 g on average across categories and in Baking Ingredients, Carbonated Drinks, Breakfast Cereals and Still Drinks. Therefore, both the proportion of sweet products categories and the absolute total sugars content of packaged foods in LMICs were reduced over the 9 years studied. These results align with recent global trends of sugar reduction in the packaged food supply (18). The reduction in total sugars in soft drinks had also previously been described in Colombia between 2016 and 2018 (35). This suggests that fewer soft drinks are being launched in LMICs following 2015, while previous studies highlight the growth in sales of sugar sweetened beverages globally (36). Purchase data analyses are required to confirm if the trends in soft drinks have actually reversed over time, or if patterns are different in LMICs compared to other countries.

Implementation of High In labels and Traffic Lights have also been associated with significant sodium reductions in LMICs where they are used. This is in agreement with what has been observed in high-income countries. High In labels in Chile, Traffic Lights in the U.K and Choices in the Netherlands have fostered reformulation of the food supply toward lower sugar and sodium (37, 38). High In labels have also been previously associated with product reformulation toward a reduction in public health sensitive nutrients in Peru and Mexico (14, 15). However, implementation of Choices, a voluntary FOPL used in South East Asia, was not associated with an improvement in sodium content, despite its proven efficacy on reformulation in the Netherlands. In general, voluntary FOPL results in lower and less consistent effects on products reformulation (39). Nonetheless, improvements observed in this study with the implementation of a FOPL suggest that food policies, such as High In labels, may assist LMICs in curbing the rise in packaged foods high in sugar, SFA and sodium and combat the nutrition transition toward a stage of high obesity and non-communicable disease prevalence (5).

In Peru, improvements in energy, protein, SFA and total sugars were significantly higher than in most other Latin American countries. Peru is the first LMIC in Latin America to have used High In labels. These were implemented in 2017 (40), 3 years before Brazil (29) and Mexico (41) and 5 years before Argentina (30) and Colombia (42). Since reductions are observed in nutrients discouraged by the FOPL, those reductions may be due to a sustained impact of High-In labels and suggests that the same trends could be observed in other Latin American countries in a few years.

Our study provides new insights into the evolution of the products sold and nutritional quality of newly launched packaged foods in 19 LMICs across 6 world regions. This information is critical to understanding the food environment in LMICs and how it has changed in the context of nutrition transition toward higher consumption of packaged foods. Furthermore, the database and statistical methods chosen for this analysis enabled the characterization of trends by country, region, product category and type of FOPL implemented. The data extracted from the Mintel GNPD spans over 9 years allowing to evaluate trends in the evolution of the packaged food supply, which could not be done using generic food composition databases. The use of Principal Components Analysis on the products sold and median nutrient content by country and category was key to identifying regional and country-specific patterns in the quality of the packaged food supply in LMICs. Additionally, analyzing the percentage of products with an improved content compared to the 2015–2017 median was necessary to assess association of FOPL with the nutritional quality of the food supply regardless of the baseline content, which differed by country and category.

However, there are some limitations to consider. The Mintel GNPD captures only launches advertised as new in a retail store. Products reformulated silently and legacy products are missing from this evaluation. In addition, new launches may not contribute the most to overall intake, so evolution in nutritional quality observed in this analysis captures the impact of FOPL on reformulation but may not reflect actual changes in nutrient intakes. Future studies could be performed on other commercial food purchase databases featuring sales such as Nielsen or Euromonitor (20) to better assess the impact of FOPL on the whole food supply.

Another limitation is that the Mintel GNPD only captures nutrient content declared on-pack, which depends on local regulations. Evolution in the percentage of products with a declared content could influence perceived nutritional quality. This is why sugar content was excluded from the analysis of the evolution of the percentage of “improved” products in Brazil and Argentina where declaration of total sugars was only made mandatory in 2021/2022. Harmonization of nutrient labeling across countries would greatly improve monitoring of packaged foods nutritional quality globally. Additionally, some nutrients relevant for public health in LMICs such as free sugars or micronutrients could not be monitored. Some studies have attempted to tackle this issue using machine learning to predict the content in free sugars of packaged foods (43). Future research may use this algorithm or alternative datasets to assess free sugar levels in packaged foods and investigate potential substitution effects between total sugars and free sugars. Notably, Labonté et al. recently demonstrated, through a French-Canadian survey, that substituting total sugars with free sugars in three nutrient profiling models had little to no effect on the association with diet quality and health markers. These findings suggest that tracking total sugars remains relevant for public health (44).

In this study, we focused on the top 20 categories in terms of new launches in Mintel GNPD to study the most relevant product categories in LMICs and avoid analyzing changes in smaller less consumed categories. This was based on the assumption that the more numerous the launches, the higher the demand for this product offering in LMICs. This methodological choice explains for instance the absence of dairy products in the analysis. Further studies could replicate the same methodology on specific food categories of interest.

Statistically significant improvements were identified in total sugar (−2.0 g/100 g) and protein content (+0.4 g/100 g) but the clinical relevance of these findings has yet to be established. Additionally, these changes were measured per 100 g, and their overall impact may be influenced by concurrent trends in portion sizes. Future research should consider examining shifts in consumer behavior and portion size across the relevant countries.

The impact of FOPL on product reformulation can be influenced by other existing national nutrition policies. For instance, food and sugar taxes, i.e., a levy applied to a certain food category or to products considered as high in a public health sensitive nutrient, may also encourage manufacturers to reduce sugar or sodium content in their products (45). Among the countries considered in this study, 10 had a food or sugar tax implemented before 2023: Mexico (2014), Ecuador (2016), Sri Lanka (2017), Peru (2018), and Malaysia (2019), all of which have FOPL, as well as India (2017), Turkey (2017), South Africa (2018), Philippines (2018), and Nigeria (2022), where there are no FOPL regulations (46, 47). The proportion of countries implementing a food tax being similar among countries with a FOPL (5 out of 10) and those without (5 out of 11), this was not considered as limitation in our study.

In conclusion, this study examines the changes in product offerings and nutritional quality of newly launched packaged food products in 19 low- and middle-income countries (LMICs) from 2015 to 2023. A shift toward more animal-based products and fewer sweet products was observed, with notable improvements in protein and total sugars content. The implementation of front-of-package labeling was further correlated with reductions in total sugars, and, depending on the type of scheme implemented, with reduction in sodium. These findings provide insights on the food environment in LMICs undergoing a nutrition transition and on certain food policies implementation is associated with reformulation of packaged foods. Future research should attempt to establish causality between food policies and food reformulation and continue to examine the broader implications of these trends and impact on dietary intakes and public health outcomes.

## Data Availability

GNPD is a commercial dataset produced and distributed by Mintel. Requests to access these datasets should be directed to https://mintel.com.
